# Supplementary material for: Early Mechanisms of Chemoresistance in Retinoblastoma
Source: Cancers (Basel). 2022 Oct 10;14(19):4966. doi: 10.3390/cancers14194966 (PMC9563111; doi:10.3390/cancers14194966)
Supplement: Supplementary file 1 [file cancers-14-04966-s001.zip › Figure S1_CarboplatinRechallenge.pdf]

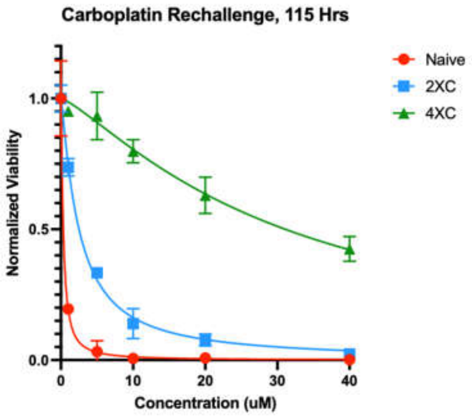

Figure S1. Carboplatin challenge performed at earlier passages demonstrates repeated sublethal carboplatin exposure induces chemoresistance in RB028 (EC_{50} for $RB028^{Naive}$ ~0.3316 μM; EC_{50} for $RB028^{2xC}$ ~2.508 μM; EC_{50} for $RB028^{4xC}$ ~31.10 μM).
