# Supplementary material for: Early Mechanisms of Chemoresistance in Retinoblastoma
Source: Cancers (Basel). 2022 Oct 10;14(19):4966. doi: 10.3390/cancers14194966 (PMC9563111; doi:10.3390/cancers14194966)
Supplement: Supplementary file 1 [file cancers-14-04966-s001.zip › Figure S2_BarcodeDiversity.pdf]

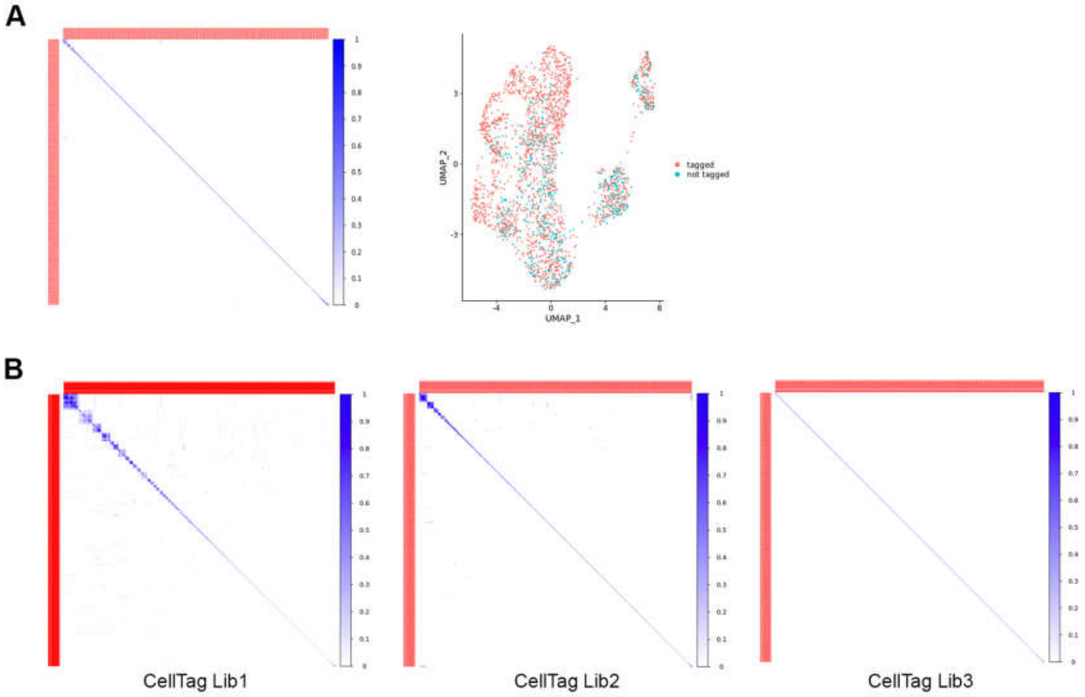

Figure S2. Assessment of CellTag barcode diversity. (**A**) Correlation plot with pairwise correlation scores and hierarchal clustering of CellTag Library 1 barcodes in RB028^{Naive} demonstrates diverse barcoding combinations (left). Clones form “blocks” representing high correlation. Lack of these blocks in RB028^{Naive} demonstrates uniquely tagged clones. UMAP of RB028^{Naive} demonstrates high percentage of tagged cells with no separation from untagged cells (right). (**B**) Correlation plots of CellTag Library 1-3 barcodes after integrating RB028^{Naive}, RB028^{2xC}, and RB028^{3xC}. Larger “blocks” formed by CellTag Library 1 barcodes represent cells sharing the same clonal origin.
