# Supplementary material for: Early Mechanisms of Chemoresistance in Retinoblastoma
Source: Cancers (Basel). 2022 Oct 10;14(19):4966. doi: 10.3390/cancers14194966 (PMC9563111; doi:10.3390/cancers14194966)
Supplement: Supplementary file 1 [file cancers-14-04966-s001.zip › Figure S3_DCA.pdf]

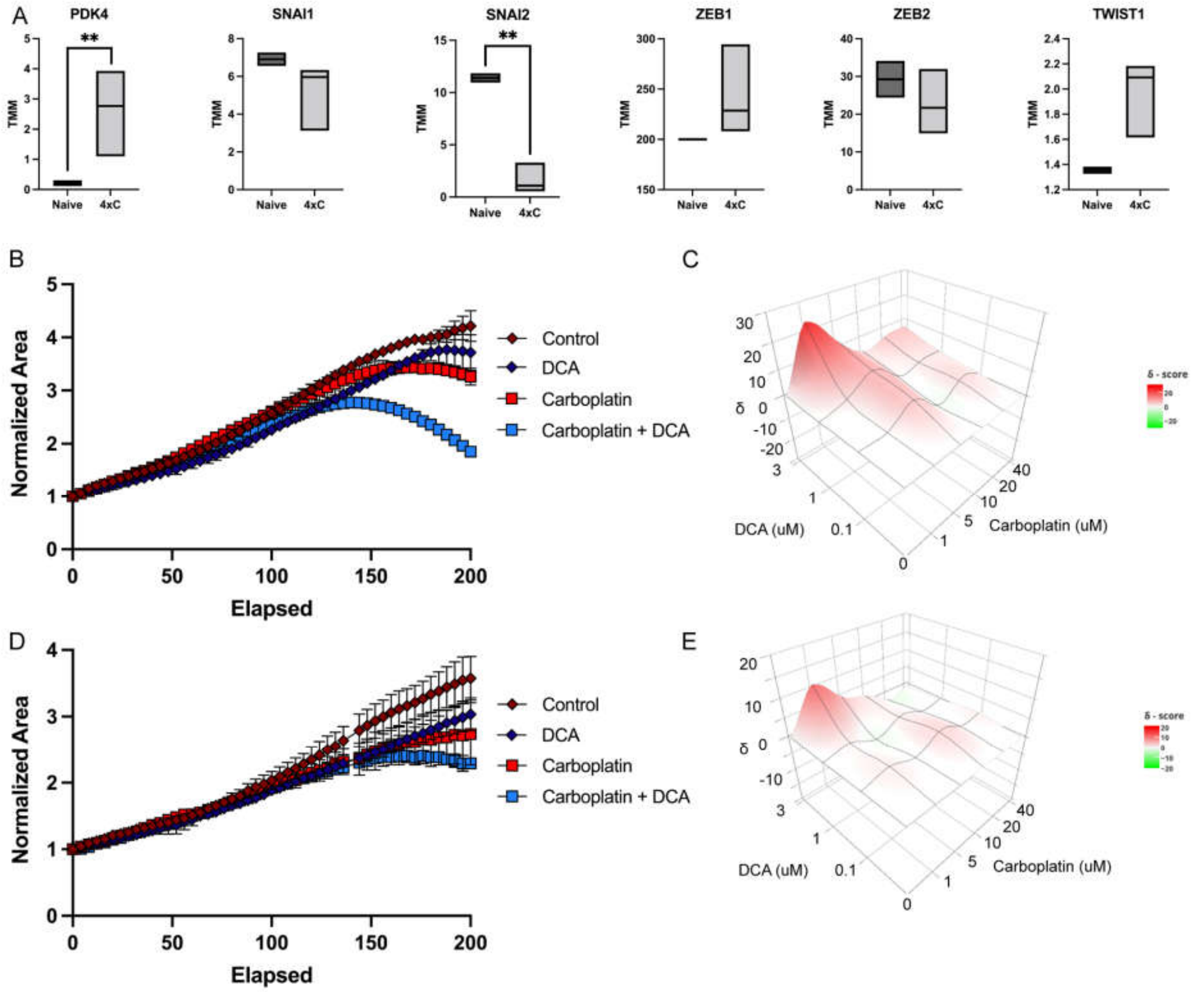

Figure S3. Effects of DCA in carboplatin chemoresistance in retinoblastoma. (**A**) Expression of key EMT transcription factors from RNAseq. (**B**) Normalized viability of RB028^{4xC} cells monitored over one week after challenge with carboplatin (1 μM) along with DCA (3 μM). (**C**) 3D synergy map of RB028^{4xC} using Bliss scores. (**D**) Normalized viability of RB006 cells monitored over one week after challenge with carboplatin (1 μM) along with DCA (3 μM). (**E**) 3D synergy map of RB006 using Bliss scores.
