# Supplementary material for: Early Mechanisms of Chemoresistance in Retinoblastoma
Source: Cancers (Basel). 2022 Oct 10;14(19):4966. doi: 10.3390/cancers14194966 (PMC9563111; doi:10.3390/cancers14194966)
Supplement: Supplementary file 1 [file cancers-14-04966-s001.zip › Figure S4_RB006Tariquidar_164.pdf]

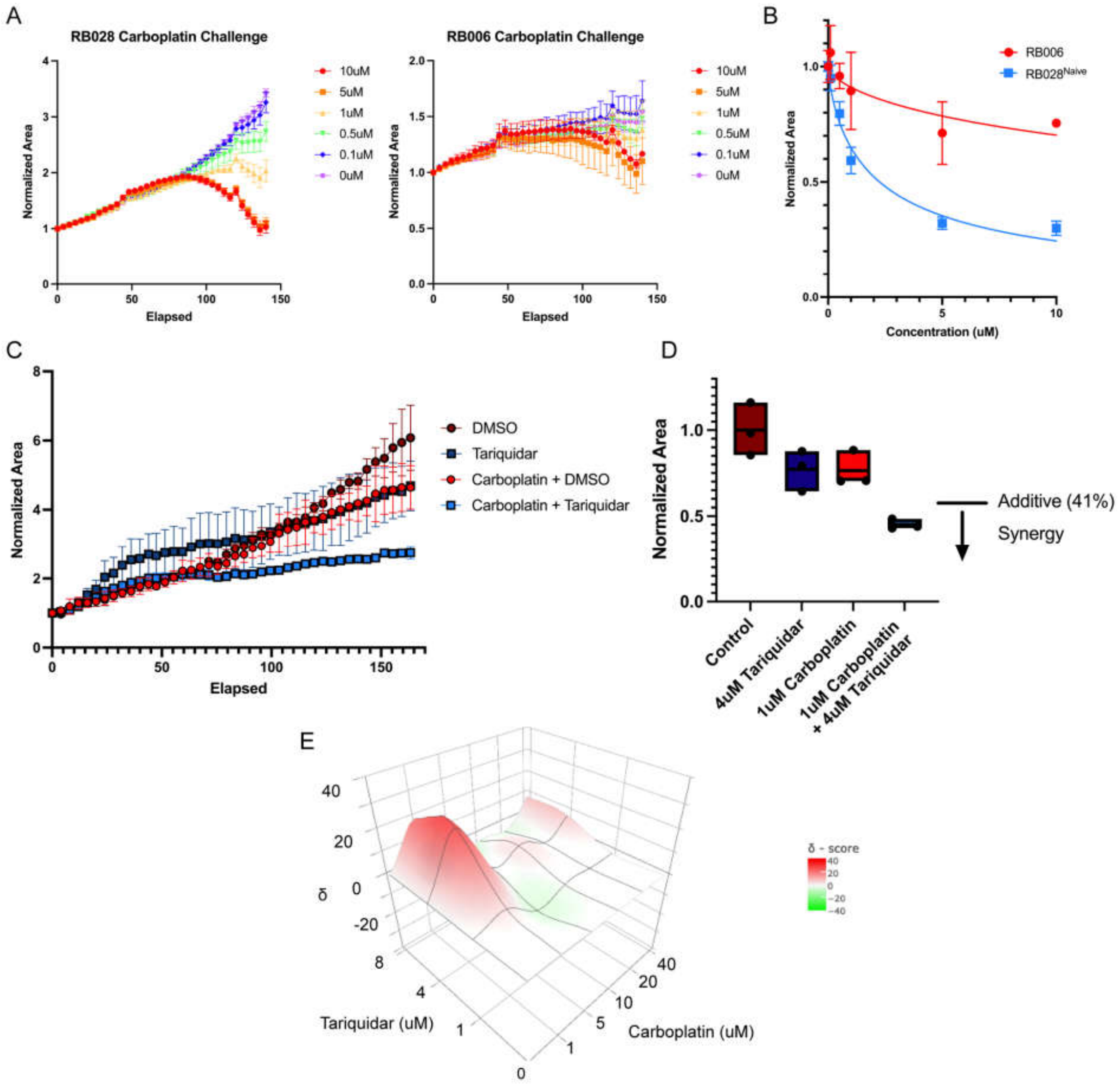

Figure S4. Carboplatin resistance potentiated by ABCB1 inhibitor tariquidar in RB006. (**A**) Carboplatin challenge of RB006 and RB028^{Naive} over one week. (**B**) Representative kill curve at Hour 140 for carboplatin challenge demonstrates greater carboplatin resistance for RB006 compared to RB028^{Naive} (EC_{50} for RB028^{Naive} ~2.26 µM; EC_{50} for RB006 ~39.04 µM). (**C**) Normalized viability of RB006 cells monitored over 1 week after challenge with carboplatin (1 µM) along with tariquidar (4 µM). (**D**) Normalized viability of RB006 cells at the 150 hour time point from panel C for the indicated treatment groups (Bliss score = 41.0%, observed inhibition = 55.8%, excess over Bliss score = 13.8%). (**E**) 3D synergy map of RB006 using Bliss scores.
