# Supplementary material for: Early Mechanisms of Chemoresistance in Retinoblastoma
Source: Cancers (Basel). 2022 Oct 10;14(19):4966. doi: 10.3390/cancers14194966 (PMC9563111; doi:10.3390/cancers14194966)
Supplement: Supplementary file 1 [file cancers-14-04966-s001.zip › Figure S5_PI3KAkt.pdf]

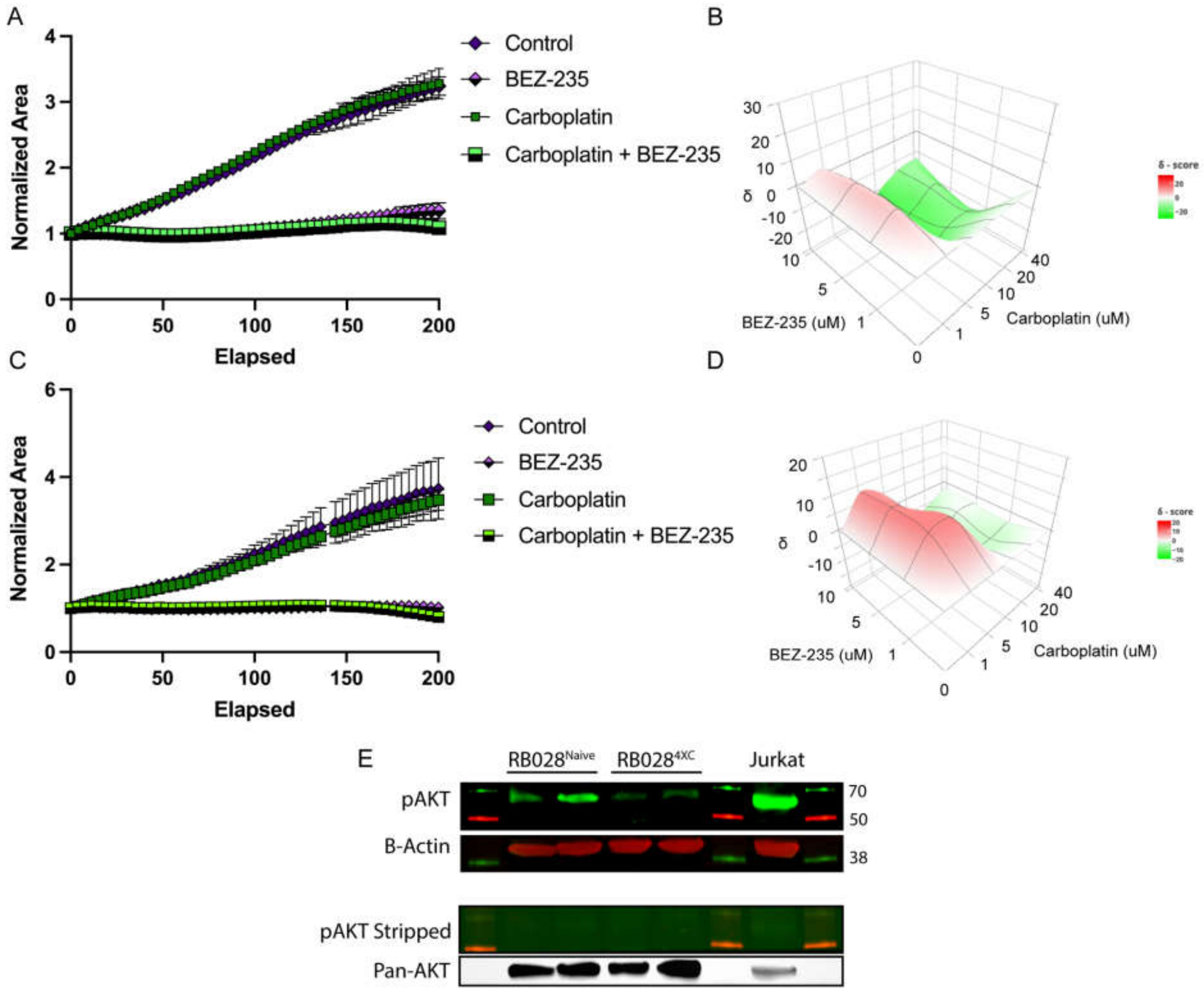

Figure S5. Effects of PI3K/AKT in carboplatin chemoresistance in retinoblastoma. (**A**) Normalized viability of RB028^{4xC} cells monitored over 10 days after challenge with carboplatin (1uM) along with BEZ-235 (1uM). (**B**) 3D synergy map of RB028^{4xC} using Bliss scores. (**C**) Normalized viability of RB006 cells monitored over 10 days after challenge with carboplatin (1uM) along with BEZ-235 (1uM). (**D**) 3D synergy map of RB006 using Bliss scores. (**E**) Western blot of pan-AKT and phosphorylated Akt (pAKT) in RB028^{Naive} and RB028^{4xC}. Jurkat cells were used as a control sample.
